# Prevalence of Musculoskeletal Dysfunctions among Indian Pregnant Women

**DOI:** 10.1155/2015/437105

**Published:** 2015-01-06

**Authors:** Preetha Ramachandra, Arun G. Maiya, Pratap Kumar, Asha Kamath

**Affiliations:** ^1^Department of Physiotherapy, School of Allied Health Sciences, Manipal University, Manipal 576104, India; ^2^Department of OBG, Kasturba Hospital, Manipal 576104, India; ^3^Department of Community Medicine, Kasturba Medical College, Manipal 576104, India

## Abstract

*Background and Objectives*. Pregnancy triggers a wide range of changes in a woman's body leading to various musculoskeletal dysfunctions. Most commonly reported musculoskeletal discomforts by pregnant women are low back pain and symphysis pubis pain. The culture and the environmental factors may influence the discomforts experienced by a pregnant woman. There is a dearth of literature in India, regarding the common musculoskeletal dysfunctions experienced by a pregnant woman, and hence this study. *Method*. A questionnaire to identify the musculoskeletal dysfunction was developed; content was validated and was translated to local languages through parallel back translation. 261 primiparous pregnant women participated in the study and filled the questionnaire in their native language. *Results*. Among the musculoskeletal dysfunctions reported by the pregnant women, 64.6% reported calf muscle cramps, 37.1% reported foot pain, and 33.7% experienced low back pain in their third trimester. In the second trimester, common musculoskeletal dysfunctions experienced by the women were that of calf pain (47.8%), low back pain (42%), and pelvic girdle pain (37%). *Conclusion*. Musculoskeletal dysfunctions and general discomforts very commonly affect the activities of daily living of pregnant women. Understanding the common discomforts during various trimesters of pregnancy will help to develop a comprehensive program for prevention and cure.

## 1. Introduction

A woman's body undergoes various changes during pregnancy which include weight gain, changes in posture, and joint and ligament laxity along with changes in musculotendinous strength [1].

Soon she realizes that her body is undergoing turmoil of events, and then the “aches” and the “pains” associated with pregnancy begin. Majority of the pregnant women do not seek medical help until the discomforts actually start interfering with their activities of daily living. Majority of these discomforts can be directly related to the physical changes that take place during pregnancy and their resultant biomechanical effects upon functional movement.

The incidence of back pain during pregnancy is relatively high and researchers worldwide have suggested that it may be between 30% and 70% [2–5]. A study on pelvic girdle relaxation in pregnancy found that 31.7% of pregnant women reported pain in the symphysis pubis [6].

In addition to the low back pain and symphysis pubis pain, a pregnant woman may also experience upper back pain, sacroiliac joint pain, muscle cramps, lower limb joint pains, foot discomforts, pedal edema, carpel tunnel syndrome, loss of balance, and falls.

Musculoskeletal dysfunctions could be influenced by the level of physical activity, cultural influences, and the environment. There has been a dearth of literature in India regarding the common musculoskeletal dysfunctions experienced by a pregnant woman throughout the trimesters.

The present study was conducted in a tertiary hospital in southern India. The study aimed at finding the musculoskeletal dysfunctions and general discomforts usually experienced by a pregnant woman across various trimesters so that it may help in the development of physical discomfort checklist and also for prescribing a custom-made exercise program to each individual.

## 2. Material and Methods

A questionnaire to identify the musculoskeletal dysfunctions was developed and it was content validated by seven experts: three from the field of Obstetrics and Gynecology and four from the field of Women's Health Physiotherapy. There were 23 items in the questionnaire which were related to musculoskeletal and general discomforts. All the items were included in the questionnaire as there was no difference in opinion among the experts (100% agreement). The questionnaire was translated to local languages and was back translated to English language. The understandability was tested by administering it to 10 pregnant women in various trimesters and after the corrections, the final questionnaire was prepared.

Anticipating 50% of pregnant women to have experienced musculoskeletal problems with a relative precision of 10% at 95% confidence level, the sample size required for screening was calculated to be 384. Primiparous women with no prior history of musculoskeletal complaints in the age group of 20 to 35 years were included in the study. A written informed consent was obtained from the participants and it was administered on pregnant women who attended antenatal physiotherapy classes between August 2011 and July 2013. The questionnaire included demographic data, parity, and specific questions related to the musculoskeletal discomforts that they experienced during pregnancy. The physiotherapist was available in case the participant had any doubt regarding the questions. Women who reported pain were further asked to mark the location of pain using a pain diagram. Areas marked above the level of the 5th lumbar vertebra (L5) were classified as low back pain; areas marked below the level of 5th lumbar vertebra and the iliac crests (anterior, posterior, and/or lateral view) were classified as pelvic girdle pain [7]. Further, in order to distinguish the pelvic girdle pain from the lumbar pain, a trained physiotherapist performed the posterior pelvic pain provocation test which has high sensitivity, specificity, and reliability [8]. Hip pain was diagnosed using FABER test [9]. All the participants completed the questionnaire and the data was analyzed using SPSS version 16.0.

## 3. Results

We had screened 384 women and 123 pregnant women were excluded from the study as they were multiparous and had already existing musculoskeletal dysfunctions prior to pregnancy. The remaining 261 pregnant women who participated in the study were primiparous women. The mean age group of the pregnant women was 27.1 ± 3.4 years (Mean ± SD).

Table 1 represents the prevalence of musculoskeletal dysfunctions in percentage (%) across various trimesters and Table 2 represents the prevalence of general discomforts in percentage (%) across various trimesters.

The results have been categorized across 3 trimesters with 30 pregnant women in their first trimester, 65 women in second trimester, and 116 women in the third trimester.

## 4. Discussion

In our study we have found that the prevalence of low back pain in first trimester is lower than the second and the third trimesters. This is in agreement with the fact that the low back pain in pregnancy is exacerbated by the softening of the ligaments and joints of the lumbosacrum in addition to the elevated levels of progesterone and relaxin hormones [3–5, 10, 11]. In our study we have observed that the amount of time a pregnant woman took to rest (Mean ± SD) 12.3 ± 2.3 hours was more in the third trimester when compared to the second trimester (Mean ± SD) 9.5 ± 1.4 hours. This could be the reason for reduced low back pain in the third trimester.

The prevalence of the upper back pain was observed only in the third trimester (3.4%). Four women who had mentioned this complaint were observed to have kyphotic posture of the upper back during postural evaluation. On further evaluation it was observed that the women did not wear supportive undergarments regularly as they believed that it caused “tightness of chest.”

In our study, the prevalence of pelvic girdle pain (PGP) was reported to be less in the third trimester (32.5%) compared to the second trimester (37%). Studies conducted in western countries have brought out that the prevalence of pregnancy-related low back pain and PGP varies between 3.90% and 89.88% [12, 13]. Major biomechanical factors associated with PGP are excessive pressure on the spine due to increased abdominal load, decreased stability in the pelvic girdle, laxity of the sacroiliac joints, and increased mobility of joints during pregnancy [9].

We also observed that 10.4% of the pregnant women experienced symphysis pubic dysfunction (SPD) in their third trimester compared to second and first trimesters in which none of the participants experienced it. The women experienced the pain especially during toileting activities, while trying to put their pants standing on one leg, while getting up from the chair, while rolling from one side of the bed to the other, and while sitting on the bed or low mat with their legs crossed. Women preferred to sit on the floor with their legs crossed (especially while eating food) as they believed that sitting in this position during third trimester may facilitate a normal vaginal delivery. In a study, pubic symphysis dysfunction has been reported in 31.7% of pregnant women across various trimesters [14]. SPD has also been reported by 12% participants in first trimester, 34% in the second trimester, and 52% in the third trimester [15]. In our study the prevalence rate could have been less due to the reduced level of physical activity during pregnancy in general.

In our study, the prevalence of coccydynia was found to be 1.5% and 1.7% in the second and the third trimesters, respectively. This could be attributed to the effect of the hormones (relaxin and progesterone) which is responsible for the ligament laxity and increased mobility of the joints [16].

Among the lower limb joint pains, we found that the pain in the knee joint was more prevalent than the ankle and the hip joint pains. Also the prevalence was more in the third trimester when compared to the second and the first trimesters. This result is in accordance with the study conducted by Bányai et al. who have detected an increased weakness of the muscles around the knee joint as the pregnancy progresses. Along with this a weaker proprioceptive perception of the anterior-posterior direction was also detected which explains the higher risk of injury of the anterior cruciate ligament. It has been reported that laxity of ligaments around the knee joint occurs during the second half of pregnancy, but it is not exacerbated by exercise programs with minimal to moderate weight-bearing [17].

Muscle cramps are very commonly experienced by pregnant women and, in our study, we have observed that the prevalence of calf muscle cramps was more than the foot muscle cramps. The prevalence was high in women in their third trimester compared to other trimesters. The cramps were due to painful muscle contractions and were generally experienced in the calf muscles at night. The prevalence of thigh muscle cramps was found to be more in the first trimester compared to other trimesters. This could be attributed to the increased symptoms of morning sickness in the first trimester which may in turn lead to dehydration and cramps.

The prevalence of neck pain was found to be 6.2% in the second trimester and less in the third trimester as all the participants who had reported neck pain were involved in use of laptop/computer for long hours 8 ± 2.1 hours (Mean ± SD).

It has been also reported that carpal tunnel syndrome is the second most common musculoskeletal complication experienced during pregnancy, the first being pain in the lumbar region [18]. This is a concern because the hands are organs of extreme importance in daily activities, especially during pregnancy and after delivery during which women need to use hands to take care of the baby, while carrying and breastfeeding. In our study we have observed that the prevalence of carpal tunnel syndrome was 9.3% in the second trimester and 5.1% in the third trimester.

Early morning stiffness of upper and lower limb joints was reported by the women only in their third trimester. The reasons behind these complaints could have been the fluid stasis and lack of blood circulation that happens after prolonged rest. Among the general discomforts the most predominantly reported complaints were that of pedal edema (36.2%), fatigue (32.8%), and urinary incontinence (20.6%). Pedal edema was more observed in women who were sedentary. The lack of lower limb muscle activity and dependent positioning of lower limbs could have been the reasons for extravasation of fluid to the extra cellular spaces, thus leading to pedal edema. The same reason could be attributed to the cause of tingling and numbness in lower extremities [16].

Fatigue was reported by women more in their third trimester than in their second trimester. Lack of sleep at night, excessive weight gain, and difficulty in breathing while lying supine could have been the causes of fatigue. All women who reported the urinary incontinence had complaints of involuntary leakage of urine during coughing and while lifting weights. Five of them also reported urge incontinence during their daily routine. Weak abdominal muscles and pelvic floor musculature could be the main reasons for urinary incontinence especially in the third trimester [19].

## 5. Conclusion

Prevalence of musculoskeletal dysfunctions and general discomforts are very common among pregnant women. But they do not report such discomforts until it affects their daily routine. Understanding the discomforts that are commonly prevalent during pregnancy will help health professionals to form a structured intervention as a part of prevention, which will in turn help the women to take care of their health during pregnancy.

## Figures and Tables

**Table 1:** Prevalence of musculoskeletal dysfunctions across trimesters in percentage (%).

Dysfunctions	First trimester (*n* = 30)	Second trimester (*n* = 65)	Third trimester (*n* = 116)
Low back pain	3.3	42	33.7
Pelvic girdle pain	3.3	37	32.5
Knee joint pain	3.3	7.7	7.8
Ankle joint pain	3.3	6.2	5.8
Hip joint pain	0	4.7	12.1
Pubic symphysis pain	0	0	10.4
Neck pain	0	6.2	2.5
Foot pain	13.3	17	37.1
Foot muscle cramps	3.3	9.3	15.1
Calf muscle cramps	26.7	47.8	64.6
Thigh muscle cramps	3.3	1.5	1.7
Tingling & numbness in lower limbs	10	15.4	4.3
Carpal tunnel syndrome	0	9.3	5.1
Stiffness in upper limb joints	0	0	7.7
Stiffness in lower limb joints	0	0	1.7
Coccydynia	0	1.5	1.7
Upper back pain	0	0	3.4
Trapezius spasm	0	1.5	1.7

**Table 2:** Prevalence of general discomforts across trimesters in percentage (%).

Discomforts	First trimester	Second trimester	Third trimester
Nausea	20	0	1.7
Vomiting	20	1.5	0
Heart burn	3.3	9.2	12.06
Fatigue	20	4.6	32.8
Pedal edema	0	29.2	36.2
Breathlessness	0	12.3	18.1
Urinary incontinence	13.3	9.2	20.6
Varicose veins	0	4.6	3.4
